# Imaging findings of thoracic manifestations of Crohn’s disease and ulcerative colitis

**DOI:** 10.1186/s13244-024-01742-4

**Published:** 2024-08-07

**Authors:** Quentin Cassius De Linval, Maxime Barat, Mathilde Aissaoui, Marie-Pauline Talabard, Clémence Martin, Georgia Malamut, Emma Canniff, Philippe Soyer, Marie-Pierre Revel, Guillaume Chassagnon

**Affiliations:** 1 Department of Radiology, Hôpital Cochin, AP-HP.Centre Université Paris Cité, 27 rue du Faubourg Saint-Jacques, 75014 Paris, France; 2 Université Paris Cité, 85 Boulevard Saint-Germain, 75006 Paris, France; 3 Respiratory Medicine and Cystic Fibrosis National Reference Center, Hôpital Cochin, AP-HP.Centre Université Paris Cité, 27 rue du Faubourg Saint-Jacques, 75014 Paris, France; 4 Department of Gastroenterology, Hôpital Cochin, AP-HP.Centre Université Paris Cité, 27 rue du Faubourg Saint-Jacques, 75014 Paris, France

**Keywords:** Inflammatory bowel diseases, Bronchial diseases, Multidetector computed tomography, Pneumonia, Tracheal diseases

## Abstract

**Abstract:**

Thoracic manifestations of inflammatory bowel disease (IBD) are rare, occurring in less than 1% of patients. Unlike most other extra-intestinal manifestations, they predominate in patients with ulcerative colitis rather than in Crohn’s disease. In most patients, thoracic involvement follows the onset of IBD by several years. However, thoracic involvement may also occur synchronously or even precede the onset of digestive symptoms. The thoracic manifestations of IBD include airway involvement and parenchymal lung abnormalities. Airways are the most frequent anatomical site for thoracic involvement in IBD. Airway manifestations usually develop several years after the onset of intestinal manifestations, preferentially when the latter are stable or in remission. Airway manifestations include bronchial wall thickening, bronchiectasis, small airway disease, and tracheal wall thickening. Parenchymal lung abnormalities are less prevalent in IBD and include organizing pneumonia, necrobiotic nodules, noncaseating granulomatous nodules, drug-induced pneumonia, and rarely interstitial lung diseases. The differential diagnosis between organizing pneumonia, necrobiotic nodules, and noncaseating granulomatous nodules is difficult and usually requires histopathological analysis for a definite diagnosis. Radiologists play a key role in the detection of thoracic manifestations of Crohn’s disease and ulcerative colitis and, therefore, need to be familiar with their imaging findings. This article aims to offer an overview of the imaging findings of thoracic manifestations in patients with Crohn’s disease or ulcerative colitis.

**Critical relevance statement:**

Thoracic manifestations of Crohn’s disease and ulcerative colitis include tracheal involvement, bronchiectasis, small airway disease, and parenchymal lung abnormalities such as organizing pneumonia and necrobiotic nodules. These rare manifestations (< 1% of patients) more often affect patients with ulcerative colitis.

**Key Points:**

- Thoracic manifestations of inflammatory bowel disease are rare, occurring in less than 1% of patients.
- Thoracic manifestations are more frequent in patients with ulcerative colitis than Crohn’s disease.
- Bronchial disease is the most frequent thoracic manifestation of Crohn’s disease and ulcerative colitis.

**Graphical Abstract:**

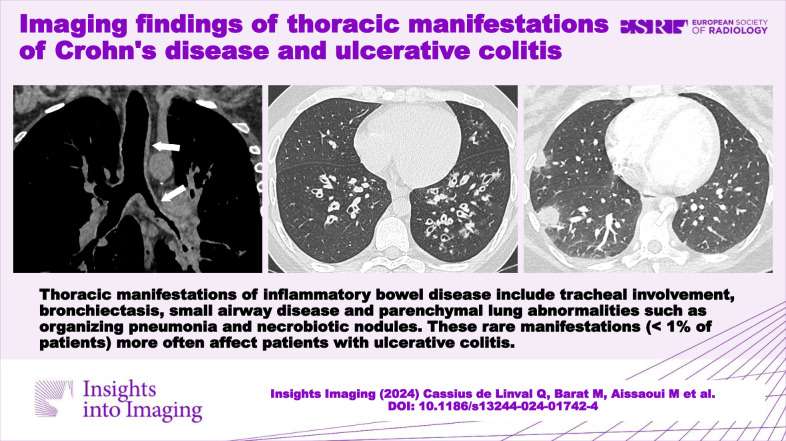

## Introduction

Crohn’s disease and ulcerative colitis are the two main causes of chronic inflammatory bowel diseases (IBD) [1, 2]. Extra-intestinal manifestations of IBD are frequent and can involve any organ, including joints, skin, eyes, liver, pancreas, and lung [3, 4]. Thoracic manifestations of IBD are rare and often unrecognized [5]. A possible association between thoracic symptoms and IBD was first suggested by Turner-Warwick et al [6], but the relationship was established by Kraft et al [7]. The prevalence of thoracic involvement in IBD is estimated to be less than 1% [3, 8], but this may be underestimated. Indeed, recent studies have reported that up to 40–60% of patients with IBD show abnormalities on pulmonary function tests (PFTs) or chest computed tomography (CT) [9, 10]. Several studies have attempted to assess the prevalence of CT abnormalities in patients with IBD [11–13], and the review by Desai et al highlighted the disparity of results, finding CT abnormalities in 22–88% of patients [8].

Unlike most other extraintestinal manifestations, thoracic involvement of IBD is more frequent in patients with ulcerative colitis [14, 15]. Bronchial disease is the most common manifestation, but the involvement of the lung parenchyma is also possible [16–18].

In most patients with IBD, thoracic involvement follows digestive disease by several years [16]. However, it may also occur synchronously or, in less than 15% of patients, precede the onset of digestive symptoms [16]. Thoracic involvement has also been described in ulcerative colitis, and most likely after curative intestinal surgery [10]. It is important to note that thoracic manifestations observed in IBD can also correspond to opportunistic infections or drug toxicity as well as specific IBD involvement [17].

Radiologists need to be aware of the thoracic manifestations of IBD in order to correctly identify them, especially when the lung bases are visible on an abdominal CT examination, and secondly to raise the possibility of IBD in the event of lung pathology. Indeed, management of lung manifestations in the course of IBD can be challenging because the possible etiologies are multiple, including infection, drug-induced or specific, with medications ranging from antibiotic to anti-inflammatory strategies, including corticosteroids. The aim of this review is to provide an overview of the imaging findings of thoracic manifestations of Crohn’s disease and ulcerative colitis.

## Airway involvement

Airways are the most frequent anatomical site for thoracic involvement in IBD [19]. This is the most prevalent and distinctive pattern of respiratory involvement in IBD as airway inflammation represents 40–63% of the clinically significant respiratory symptoms [16].

The respiratory tract is classically divided into upper airways (pharyngo-larynx and trachea), bronchi, and small airways. Tracheal and bronchial involvement share clinical-radiological similarities. Airway involvement mainly includes tracheobronchitis with or without stenosis, chronic bronchitis, bronchial dilatation, and bronchiolitis [20].

### Upper airway disorders

Upper airway disorders include inflammation of the laryngeal region and trachea, with or without stenosis. Upper airway disorders are very rare [21–23] and predominate in males [20].

It occurs most frequently in patients with ulcerative colitis [14], with the delay between diagnosis of IBD and the onset of tracheal involvement varying from 10 to 30 years in the literature [22–24]. Tracheobronchitis as the initial presentation of IBD, has been reported in patients with ulcerative colitis [25]. In patients with ulcerative colitis, tracheal involvement can sometimes develop after colectomy [20, 23], with delays ranging from 30 days to several years or even decades.

In their literature review, Black et al pooled data from 155 patients with IBD and respiratory manifestations [14]. They found that 15 patients had upper airway involvement, with 13 experiencing tracheal lesions and two having laryngeal lesions [14]. Among these patients, 60% were men and 73% had ulcerative colitis. Clinically, the most common symptoms were cough, dyspnea, stridor, and hoarseness, with respiratory distress occurring in cases of severe stenosis. Pulmonary function tests (PFTs), when conducted, typically reveal an obstructive lung disease pattern with a normal carbon monoxide transfer coefficient (DLCO) [24, 26].

Anatomical pathology studies showed chronic mucosal and submucosal lymphoplasmacytic inflammatory infiltrates and fibroexudative debris with squamous metaplasia in both diseases [26]. In ulcerative colitis, mucosal ulcerations and microabscesses are more frequently observed, whereas in Crohn’s disease non-caseating epithelioid granulomas are suggestive but non-specific findings [27].

**Fig. 1:**
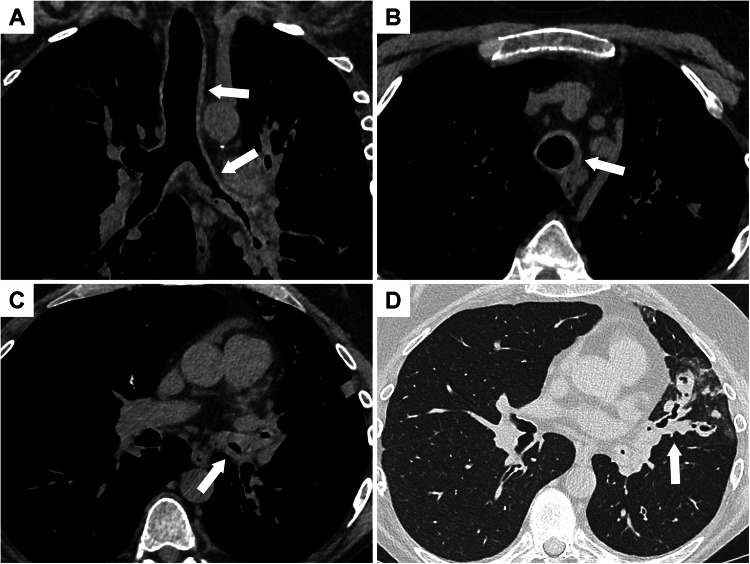
Chest CT showing airway involvement in a 48-year-old patient with ulcerative colitis diagnosed 25 years before. **A** CT image in the coronal plane shows tracheal and bronchial wall thickening (arrows). Axial images show tracheal wall thickening sparing the posterior membrane (**B**) and bronchial wall thickening of the left lower lobe bronchus with stenosis (**C**). **D** These lesions are associated with bronchial dilatation and mucus impactions (arrow) in the left upper lobe

CT imaging findings are not specific, and the most common one is circumferential wall thickening which may extend to the bronchi (Fig. 1). Tracheal wall thickening can be irregular, sometimes nodular, and does not spare the membranous posterior wall [26]. Infiltration of peri-tracheal fat is often associated. The main differential diagnoses to consider are granulomatosis with polyangiitis, relapsing polychondritis, tuberculosis, sarcoidosis, and amyloidosis.

### Bronchial disease

Bronchi are the most common locations of airway involvement in IBD [5, 15, 20]. Bronchial disease accounts for 39% of thoracic manifestations, and is thought to be responsible for half of all respiratory symptoms observed in IBD, including cough, sputum, and dyspnea [14, 20].

Bronchial involvement occurs more frequently in non-smoking women, and there is a stronger association with ulcerative colitis than with Crohn’s disease [14]. The age of onset for bronchial involvement is around the fifth decade, and does not correlate with intestinal disease activity. In the literature review of Black et al, 89% of patients had ulcerative colitis, and 81% were nonsmokers [14]. Camus et al found that in 85% of patients, the onset of bronchial disease followed gastrointestinal manifestations after several years, whereas in 5 to 10% of patients, bronchial disease manifested during the same timeframe than gastrointestinal manifestations, and in the remaining 10 to 15% of patients bronchial disease preceded gastrointestinal manifestations [16]. In the latter setting, patients were younger with a mean age of 13 ± 7.5 (standard deviation) years [16, 20]. Finally, in 79% of patients, the intestinal disease was quiescent at the time of diagnosis [14, 16, 20].

Clinically, proximal airway involvement is characterized by chronic bronchitis, which is found in almost 20% of patients [14].

**Fig. 2:**
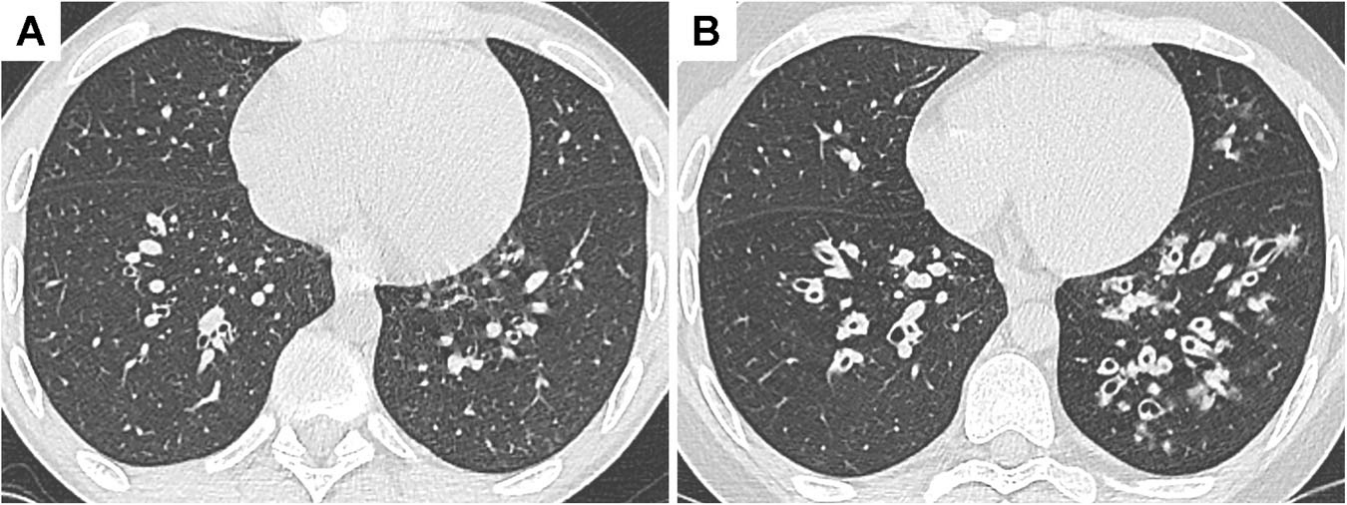
Airway disease in a patient with severe Crohn’s disease. **A** 18-year-old developed a cough a few months after colectomy. A chest CT scan performed at that time showed only slight bronchial wall thickening in the lower lobes. **B** Seven years later, chest CT shows bilateral bronchiectasis in the lower lobes with severe bronchial wall thickening

CT findings of bronchial involvement include bronchial wall thickening, bronchial stenosis, mucus plugs, and bronchiectasis (Figs. 1–2**)**. Bronchiectasis or bronchial dilatation is the most frequent thoracic manifestation of IBD [14, 20] and seems to be more frequent in ulcerative colitis than in Crohn’s disease. In a large population-based cohort study, patients with ulcerative colitis had a 66% lower age/sex-adjusted risk for bronchiectasis compared to those with Crohn’s disease [28]. In the literature review by Black et al which included 155 patients, bronchiectasis or bronchial dilatation accounted for 66% of bronchial manifestations and for nearly 30% of all abnormalities on thoracic CT examination [14].

On CT, bronchial dilatation is defined as the presence of a bronchus with an endoluminal diameter > 1–1.5 times the diameter of the adjacent artery [29].

### Small airway disease

Small airway disease accounts for 3–10% of IBD-related pulmonary manifestations, but its prevalence is probably underestimated [14, 30]. In contrast to larger airway involvement, it appears earlier in the disease course and thus at a younger age. In almost one-third of patients, it precedes the onset of digestive disease [16, 30]. In addition, the prevalence of small airway disease in ulcerative colitis is similar to that of Crohn’s disease, whereas other types of airway manifestations are more prevalent in ulcerative colitis [14].

Clinically, small airway involvement is often asymptomatic or pauci symptomatic [4]. It may manifest as a non-productive cough and dyspnea [16]. PFTs may show small airway disease, which can be associated with decreased DLCO [13]. Tzanakis et al showed that the volume of equal flows (Visov) was impaired in patients with ulcerative colitis and Crohn’s disease in contrast with controls [13]. In the latter study, Gupta et al described abnormal PFTs among 51 out of 83 patients with ulcerative colitis in remission vs. eight out of 48 controls with small airway disease impairment (defined as ppFEV_1_ < 70%, FEV_1_/FVC ratio < 70% and reduced MEF 25–75%) in 21.56% of subjects with ulcerative colitis in remission, second most frequent PFT abnormal pattern after the restrictive pattern [31].

Regarding pathological findings, different types of bronchiolitis can be observed, the most common being granulomatous bronchiolitis, which is only seen in Crohn’s disease [32]. It corresponds to a peribronchiolar infiltration of epithelioid granulomas without caseous necrosis [33]. Other forms of bronchiolitis have been described, notably in ulcerative colitis patients, such as constrictive bronchiolitis or acute necrotizing bronchiolitis [30]. Peribronchiolar lymphoplasmacytic inflammatory infiltrates and areas of small airway fibrosis have also been reported [14].

**Fig. 3:**
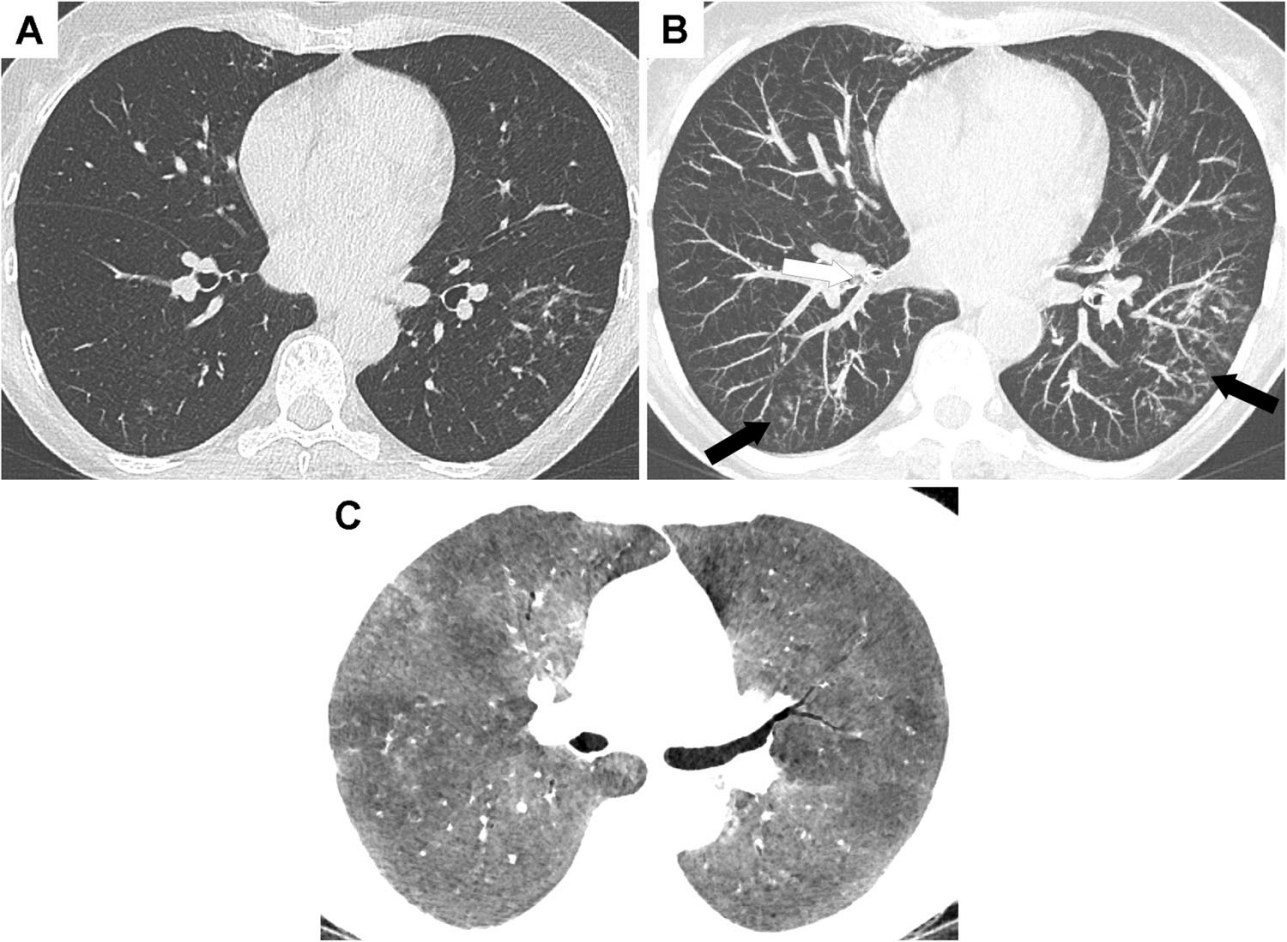
Small airways disease in a 60-year-old patient with ulcerative colitis. **A** Axial CT images show centrilobular nodules predominating in the right lower lobe. **B** Maximum intensity projection helps detect bilateral centrilobular nodules (arrows), which have a tree-in-bud appearance. **C** Minimum intensity projection shows the heterogeneity in the lung attenuation, which is related to diffuse mosaic perfusion pattern

Chest CT is the reference examination for imaging small airway disease. CT features of small airways involvement include manifestations of cellular bronchiolitis such as centrilobular nodules, frequently in a “tree-in-bud” pattern [14, 21, 34], and the manifestation of small airway obliteration, which is the presence of mosaic perfusion (Fig. 3). Mosaic attenuation pattern corresponds to the presence of areas of lesser attenuation due to small airway disease together with the areas of normal lung parenchyma. The detection of mosaic perfusion is enhanced using minimum-intensity projection reconstructions. When additional expiratory images are acquired, air trapping is observed in areas of mosaic perfusion. Bronchiolectasis can also be seen in chronic bronchiolitis [35], and signs of bronchial disease are frequently associated. These CT findings can be found in other small airway diseases, such as infectious bronchiolitis, which usually manifest as a cellular bronchiolitis and is the main differential diagnosis.

## Parenchymal lung abnormalities

The two most common parenchymal disorders associated with IBD are organizing pneumonia and necrobiotic nodules [14]. Nonspecific interstitial lung disease (NSIP) and eosinophilic pneumonia have been described, but are most often related to drug toxicity [5].

Parenchymal lung abnormalities in IBD occur most frequently in patients with ulcerative colitis, with a discrete female predominance [14, 30]. In a review by Black et al, among 155 patients with IBD and thoracic involvement, 58% were women, and 65% had ulcerative colitis [14]. Parenchymal lung abnormalities associated with IBD typically appear several years after the onset of intestinal manifestations, and their evolution is independent from that of the latter [36].

### Organizing pneumonia

Organizing pneumonia is the most common parenchymal lung abnormality in IBD, and occurs more frequently in patients with ulcerative colitis than in those with Crohn’s disease [14, 15, 21]. In an analysis of more than 400 patients with pulmonary manifestations of IBD, Storch et al found nine patients with organizing pneumonia, of which eight had ulcerative colitis [37]. In Crohn’s disease, non-caseating granulomatous infiltrates may be combined with organizing pneumonia [33]. Organizing pneumonia can be related to IBD or drug-induced [38].

Clinically, organizing pneumonia can present as a condition with fever, cough, dyspnea, and raised inflammatory markers. Organizing pneumonia is usually reversible with treatment without sequelae but tends to recur.

**Fig. 4:**
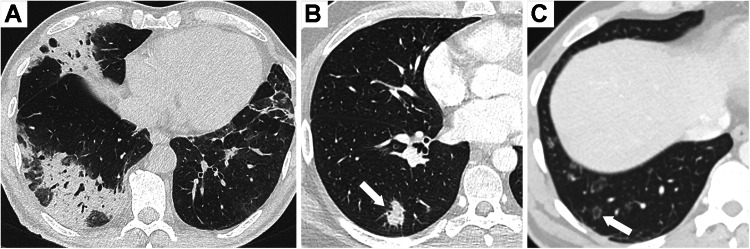
Various CT patterns of inflammatory bowel disease-related organizing pneumonia. **A** CT image in the axial plane shows bilateral consolidation with air bronchogram in a 71-year-old patient with Crohn’s disease. **B** CT image in the axial plane shows a pseudo-tumoral lesion (arrow) in a 36-year-old patient with Crohn’s disease. **C** CT image in the axial plane shows multiple consolidations with the atoll sign (arrow) in a 35-year-old patient with Crohn’s disease

**Fig. 5:**
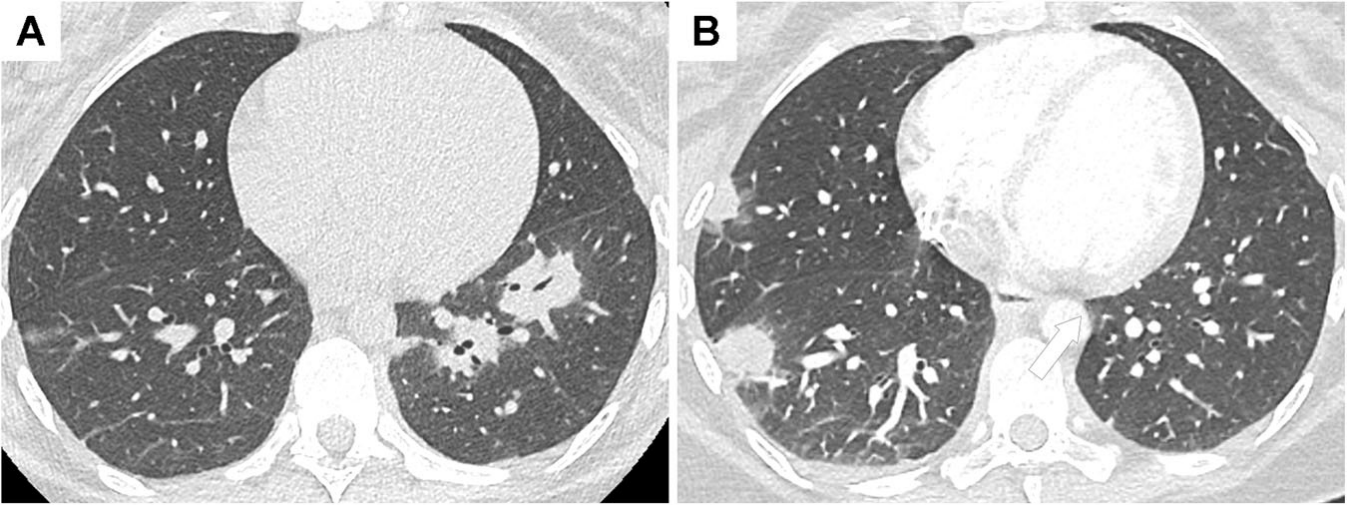
Recurrent organizing pneumonia in a 28-year-old patient with Crohn’s disease. **A** CT image in the axial plane shows peribronchovascular consolidations in the left lower lobe consistent with organizing pneumonia. These lesions disappeared with corticosteroids. **B** Few months after corticosteroids withdrawal, a recurrence of organizing pneumonia was observed in other lung areas

On CT, organizing pneumonia usually presents as multifocal alveolar consolidation, often accompanied by an air bronchogram [39]. Lesions preferentially have a subpleural or peribronchial distribution predominating in the lower lobes [39] (Fig. 4). These consolidations are nonspecific and mimic infectious pneumonia. The recurrence of consolidations in a different locations (migratory pattern) and the reversed halo sign are key imaging findings for the diagnosis (Fig. 5). The reversed halo sign, also known as the Atoll sign, was first described in patients with cryptogenic organizing pneumonia [40]. It is found in less than 20% of patients with IBD-related organizing pneumonia. It is characterized by a central ground-glass opacity surrounded by a crescentic or ring-shaped consolidation. Initially considered highly suggestive of organizing pneumonia, it has been described in several other inflammatory diseases (lupus, rheumatoid arthritis, granulomatosis with polyangiitis), infectious (fungal), vascular, and neoplastic diseases [41].

Organizing pneumonia may also be present as a single or as multiple pulmonary nodules (focal nodular organizing pneumonia) [42]. The differential diagnoses in patients with IBD include necrobiotic and noncaseating granulomatous nodules.

### Necrobiotic nodules and non-caseating granulomatous nodules

Necrobiotic nodules associated with IBD were first described in 2000 by Faller et al [43]. They account for around 5% of pulmonary manifestations encountered in IBD [16, 33]. They can be observed in both Crohn’s disease and ulcerative colitis, but recent data suggest a greater prevalence in Crohn’s disease [44, 45]. In 2012, Barbosa et al reported 15 published cases of necrobiotic nodules, 10 of which were observed in patients with Crohn’s disease [45]. The majority of cases were associated with active intestinal disease [46–48] and observed in young patients (< 30-year-old) [46]. More rarely, true granulomatous pulmonary nodules without caseous necrosis have been reported and almost exclusively in pediatric patients with Crohn’s disease [49, 50]. In a case report by Roblin et al the patient was 13-year-old and the pulmonary granulomatous involvement preceded the digestive disease, which occurred a few weeks later [49].

Histologically, necrobiotic nodules combine sterile aggregates of neutrophils with areas of central necrosis, and are closely related to pulmonary nodules in rheumatoid arthritis and cutaneous nodules in pyoderma gangrenosum [3]. They may display a pseudo-granulomatous appearance when epithelioid histiocytes are associated with aggregates of plasma cells [51]. Differentiation between necrobiotic nodules, caseous granulomas and nodular organizing pneumonia requires histological sampling, which is often not performed as it is not necessary for patient management.

The clinical presentation of necrobiotic nodules and non-caseating granulomatous nodules is nonspecific. The most frequent symptoms are dyspnea, cough and sometimes chest pain in the case of subpleural localization; fever is less frequent compared to organizing pneumonia [41].

**Fig. 6:**
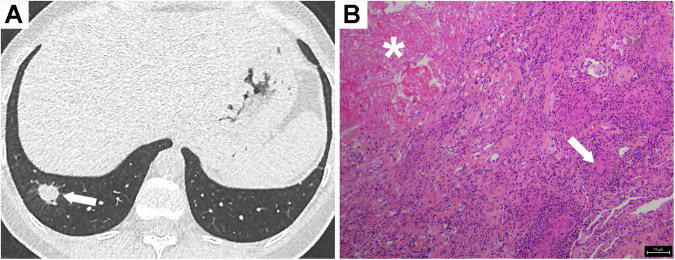
Necrobiotic nodule (histologically confirmed) in a 41-year-old patient with Crohn’s disease. **A** CT image in the axial plane reveals a right lower lobe nodule that was diagnosed during the initial Crohn’s disease flare-up and surgical resection. **B** HES staining of the lesion showing necrotizing granuloma. The central necrosis (*) is surrounded by epithelioid granuloma with giant cell (arrow)

On imaging, necrobiotic nodules and granulomatous nodules have a similar presentation. On CT, they are solid nodules or masses generally measuring less than 3 cm, are predominantly located in subpleural areas, and may subsequently cavitate (Fig. 6) [45, 46, 52]. Due to their nonspecific appearance, there is no criterion for differentiating these two types of lesions. Granulomatous nodules may also present as consolidations, mimicking organizing pneumonia.

### Interstitial lung diseases

**Fig. 7:**
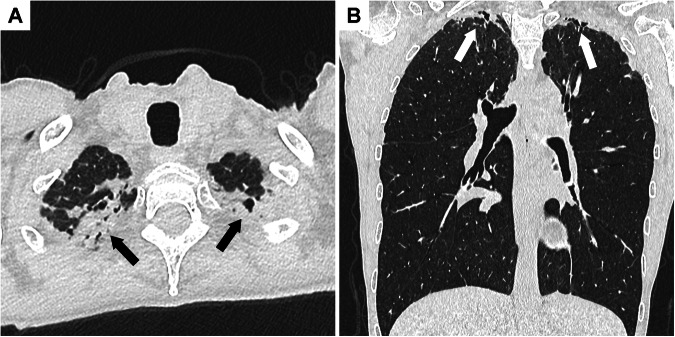
Pleuroparenchymal fibroelastosis in a 70-year-old patient with ulcerative colitis. **A** CT image in the axial plane shows bilateral subpleural consolidations with bronchiolectasis. **B** CT image in the coronal plane shows apical distribution and volume loss in the upper lobes. The combination of these CT findings is highly suggestive of the diagnosis of pleuroparenchymal fibroelastosis, and no surgical biopsy has been performed

Interstitial lung diseases (ILDs) are more frequently associated with ulcerative colitis than with Crohn’s disease. A recent literature review identified 31 patients with ILD, the majority of which had ulcerative colitis (22/31), and 64% were drug-induced [53]. ILDs other than organizing pneumonia that have been reported in patients with IBD include pleuroparenchymal fibroelastosis (Fig. 7), NSIP, desquamative interstitial pneumonia, eosinophilic pneumonia, and usual interstitial pneumonia [14, 53–55]. These ILD are less common than organizing pneumonia, which represents almost half of the cases of ILD observed in IBD patients [53].

ILD usually occurs some months or even years after the onset of IBD. In a literature review on ILDs associated with ulcerative colitis, Xu et al found that the ILD occurred 1‑ to 15 years after the diagnosis of ulcerative colitis in 12 patients (12/14, 85%), and five of them developed ILD during IBD activity (5/12; 42%) [56].

### Drug-induced pneumonias

**Fig. 8:**
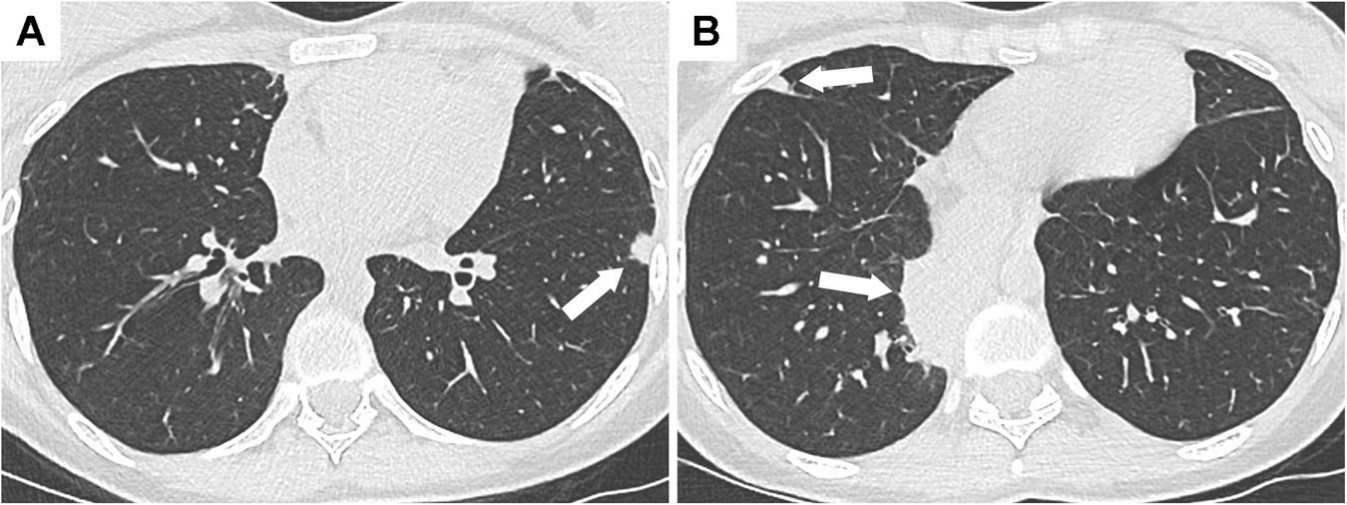
Drug-induced pneumonia in an 18-year-old patient with ulcerative colitis treated with mesalamine. CT image in the axial plane images show bilateral subpleural consolidations (arrows in **A** and **B**). CT-guided transthoracic biopsy was performed and showed pathological findings compatible with mesalamine-induced lung disease

In IBD, drug-induced pneumonias are mainly related to purine analogues (Azathioprine, 6-mercaptopurine), 5-ASA derivates (mesalazine, sulfasalazine), Methotrexate, and anti-tumor necrosis factor (TNF) alpha [53, 57, 58]. The clinical presentation is non-specific with the most frequent symptoms being dyspnea, cough and fever, however patients can be asymptomatic [53]. On imaging, drug-induced pneumonias can show different patterns, such as organizing pneumonia (Fig. 8) or nonspecific interstitial pneumonia [16, 43, 59]. The diagnosis of drug-induced pneumonia is based on (i), a similar radiological and/or histopathological pattern, (ii), exclusion of other etiologies, (iii), improvement after discontinuation of treatment and, (iv), relapse of symptoms in cases of drug rechallenge. Anti-TNF alpha used in treatment can also cause infectious complications, including tuberculosis.

### Infectious pneumonias

IBD are also associated with an increased risk of infectious pneumonia, even in untreated patients. In a cohort of 74,156 IBD patients and 1,482,363 controls without IBD, Kantsø et al. showed that Crohn’s disease and ulcerative colitis are associated with an increased risk of invasive pneumococcal disease before and after the diagnosis of IBD (hazard ratio, 1.99; 95% confidence interval, 1.59–2.49 for Crohn’s disease and 1.46; 95% confidence, 1.25–1.69 for ulcerative colitis). Pneumococcal vaccination is therefore recommended for all IBD patients [60].

The use of immunosuppressive agents also increases the risk of opportunistic pulmonary infection. Immunosuppressive agents used in IBD include systemic corticosteroids, anti-TNF agents, and JAK inhibitors. Biologic therapies and JAK inhibitors are associated with an increased risk of tuberculosis. For this reason, IBD patients should be screened for latent tuberculosis prior to immunosuppression [61]. Screening for latent tuberculosis is based on a combination of epidemiological risk factors, physical examination, chest X-ray and tuberculin skin test or interferon-gamma release test. Tuberculosis is not the only pulmonary infection at increased risk under immunosuppressive therapy in IBD patients. In the event of infectious pneumonia in an IBD patient undergoing immunosuppressive treatment, the presence of Legionella pneumophila should always be suspected and the patient tested [61]. Immunosuppressive drugs also increase the risk of pneumocystis, and prophylaxis should be considered for certain treatment combinations [61].

### Differential diagnoses

The imputability of IBD in the development of thoracic lesions is sometimes difficult to assess, particularly in smokers who may smoking-related chronic obstructive pulmonary disease. IBD may also be associated with other immune disorders, which are themselves responsible for thoracic manifestations such as common variable immunodeficiency.

## Conclusion

With the increased use of chest CT, pulmonary manifestations of ulcerative colitis and Crohn’s disease are more commonly encountered. Unlike most other extra-intestinal manifestations, thoracic involvement of IBD predominates in patients with ulcerative colitis, and bronchial disease—especially bronchiectasis—is the most frequent manifestation, though parenchymal involvement is also possible. Good knowledge of these thoracic manifestations associated with ulcerative colitis and Crohn’s disease is critical for the radiologist, who plays a key role in their detection.

## Data Availability

The data of cases in the manuscript are available from the corresponding author on reasonable request.

## Abbreviations

CT: Computed tomography
DLCO: Carbon monoxide transfer coefficient
IBD: Inflammatory bowel disease
ILD: Interstitial lung disease
NSIP: Nonspecific interstitial pneumonia
PFTs: Pulmonary function tests
TNF: Tumor necrosis factor
